# Supplementary material for: Sensory adaptation and pupil-linked arousal support flexible evidence accumulation during perceptual decision making
Source: eLife. 2026 Jul 27;15:RP110685. doi: 10.7554/eLife.110685 (PMC13405624; doi:10.7554/eLife.110685)
Supplement: Peer review history

# Editor report: eLife Assessment

**DOI:** 10.7554/eLife.110685.3.sa0

**Authors:** Redmond G O'Connell

**Affiliations:** Trinity College Dublin Ireland

**Keywords:** Compelling, Important

This **important** study measures single-unit activity in area MT of awake-behaving monkeys to test the idea that sensory adaptation contributes to flexible evidence accumulation during decision making. The authors provide **compelling** evidence that adaptation to different temporal contexts shapes both perceptual judgements and neural responses. Although the precise computational mechanisms underlying these effects remain uncertain, the results support the conclusion that recent sensory history influences the temporal dynamics of decision formation. This work will be of interest to researchers studying visual perception, sensory adaptation, and decision making.

# Referee report: Reviewer #1 (Public review)

**DOI:** 10.7554/eLife.110685.3.sa1

McGaughey and Gold ask where in the decision process the flexibility of evidence accumulation arises, proposing that it is not solely a property of downstream integrators but is also supported by stimulus-specific sensory adaptation in the middle temporal area (MT). Recording single-unit activity in rhesus macaques during a motion direction-discrimination task in which an adapting stimulus of varying temporal stability precedes an identical test stimulus, they find that more rapidly changing contexts produce weaker and less discriminable MT responses to the test stimulus, which they argue accounts in part for context-dependent changes in decision-making behavior. Through session-level correlations they further identify pupil-linked arousal as a parallel, apparently separable contributor.

The main strength is the shift of perspective toward the encoding stage: rather than treating MT as a static input to flexible downstream integrators, the authors show that early sensory cortex can itself contribute adaptive, context-dependent signals that shape behavior. The conceptual advance is supported by a well-designed paradigm-total exposure to each motion direction is matched across conditions and the test stimulus is held identical-together with single-unit recordings and simultaneous pupillometry. The behavioral effect is consistent across three animals, and the fact that context-dependent differences emerge over repeated stimulus presentations within a trial, rather than as a sustained baseline offset across blocks, ties the effect convincingly to stimulus-specific adaptation.

The behavioral effect constrains the temporal dynamics of decision formation but does not uniquely identify its algorithmic basis: a leak, a saturating non-linearity, or a reduction in the gain of integration are all compatible with a shallower rise of accuracy with viewing time, and the reduced MT discriminability is itself an encoding-stage efficiency effect of this kind. The manuscript appropriately treats the algorithmic basis as unresolved, noting that distinguishing these accounts would require analyses not available here, such as reverse-correlation or motion-energy kernels with lower-coherence test stimuli.

The inference that the adaptation- and arousal-related signals operate independently rests on the absence of session-wise correlations between the neural and pupil measures and their behavioral contributions. Given the noise in the trial-wise estimates, this is best read as consistent with, rather than demonstrating, true independence, as the authors note.

Overall, the authors largely achieve their aim of showing that sensory adaptation in MT shapes the evidence available for time-dependent perceptual decisions. The evidence for a sensory-encoding contribution is convincing, while the claim of independence between adaptation and arousal is more tentative and is framed as such.

# Referee report: Reviewer #2 (Public review)

**DOI:** 10.7554/eLife.110685.3.sa2

McGaughey and Gold trained rhesus macaque monkeys to perform a motion-direction discrimination task in which a behaviorally irrelevant adapting stimulus with either fast or slow direction alternations preceded a variable-duration test stimulus, while simultaneously recording single-unit activity in area MT and pupil diameter. They report that adaptation to the more rapidly changing stimulus was associated with reduced behavioral sensitivity, attenuated test-evoked MT responses, and larger pupil-linked arousal signals. The authors interpret these behavioral changes as evidence for context-dependent adjustments to the temporal dynamics of decision formation and argue that these adjustments are supported by both sensory adaptation in MT and arousal-related mechanisms. More broadly, they conclude that flexible evidence accumulation in dynamic environments arises from distributed adjustments across sensory encoding and neuromodulatory systems rather than solely from changes within a downstream accumulator. If correct, this interpretation has important implications not only for our understanding of perceptual decision making, but also for broader theories concerning the functional role of sensory adaptation.

The conclusions of the paper are generally supported by the data. Evidence for adaptation-induced changes in sensory encoding, behavior, and pupil dynamics is convincing, and the revised manuscript substantially strengthens the connection between the behavioral findings and the proposed decision-making framework.

Comments on revised version.

The revised manuscript provides a clearer account of how recent stimulus history influences behavioral performance. In the original version, aspects of the psychometric functions were interpreted as evidence for a more leaky evidence-accumulation process, although some of these effects could potentially have reflected alternative mechanisms, including influences of the adapting stimulus on short-duration trials. The additional analyses and discussion included in the revision clarify that information from the adapting stimulus contributes to behavior at short viewing durations and appropriately temper claims regarding the specific computational mechanism underlying the observed behavioral effects. While the data do not uniquely identify whether these effects arise from changes in leak, other nonlinearities, or related decision processes, they provide convincing evidence that recent temporal context influences the temporal dynamics of decision formation.

My original review also noted that different sections of the manuscript relied on different behavioral metrics and analytical approaches when relating behavioral changes to neural and pupil-linked measures. The revised manuscript now provides a clearer rationale for these choices, including distinctions arising from the different trial types and time windows used in the neural and pupil analyses.

# Author comment: Author response

**DOI:** 10.7554/eLife.110685.3.sa3

**Authors:** Kara D McGaughey, Joshua I Gold

**Affiliations:** University of Pennsylvania Philadelphia United States; University of Pennsylvania Philadelphia United States

The following is the authors’ response to the original reviews.

**Reviewer #1 (Public review):**

(1) Alternative mechanisms for performance differences.

The authors assume that the difference in performance between the low-switch (LS) and high-switch (HS) frequency conditions is explained by a change in the "leakiness" of integration. However, several other mechanisms could potentially explain this effect:

(1) Temporal Uncertainty: Integration might start later in the HS condition, leading to lower performance.

(2) Reduced Efficiency: Integration could be less efficient in the HS condition (i.e., lower signal-to-noise ratio) without a change in the leak parameter itself.

(3) Evidence Contamination: Motion information from the adapting stimulus in the HS condition may be integrated rather than ignored, which might be the case since the transition from the adapting to the test stimulus is not externally cued.

To distinguish between these alternatives, I suggest two possible analyses. First, a formal model comparison could be performed, though I acknowledge this may be inconclusive in the absence of response-time data. Second, an analysis of motion energy kernels could be revealing; the leak hypothesis makes the specific prediction that for long test stimuli, early samples should contribute more to the choice in the LS condition than in the HS condition, relative to late samples.

We thank the reviewer for raising these important points. We agree that we cannot definitively identify the algorithmic underpinnings of the behavioral effects we report and have made substantial revisions to the manuscript to be clearer about what is supported and what is speculative in our claims. Most importantly, we agree that we do not know if the context-dependent differences in how accuracy depends on viewing time are based on adjustments to a leak or to something else (e.g., a saturating non-linearity, as we identified in Glaze et al, 2015, that is separate from the leak itself), which we cannot resolve with this dataset, even with more formal model comparisons. We therefore:

Changed the wording throughout the manuscript to refer to changes in leakiness as just one of several possible sources of the behavioral differences. We also added this point to the list of “limitations” (and possible future directions, including using motion-energy kernels, which would require us to use lower-coherence test stimuli) in the Discussion (L487-493).

Added a new figure panel (Fig. 2D), a new Extended Data figure (Extended Data Fig. 3), and additional explanatory text (L168-175) that collectively describe the behavior in more detail, including quantifying a “crossover” dynamic similar to what we reported previously (Glaze et al, 2015).

Added new explanations (L152-163) and analyses (Extended Data Fig. 9) indicating that the monkeys used some information from the end of the adapting stimulus to inform their decisions, which accounts for the patterns of choices at the shortest viewing durations.

Indicate that the context-dependent differences in the slopes of the psychometric functions (and complementary analyses based on “raw” accuracy measures as a function of binned viewing duration) rule out the temporal uncertainty and evidence contamination explanations, but are consistent with effects on the temporal dynamics of the decision process (L175-179).

(2) Independence of neural and pupil-linked signals.

The authors take the lack of session-wise correlation between context-dependent contributions from neural and pupil terms as evidence that these two signals provide independent contributions to the behavioral effect. However, could this lack of correlation simply be a result of high variability or noise in these estimates? The data shown in Figure 7B suggests that measurements are very noisy, which might obscure a potential relationship.

We agree that the lack of session-wise correlation between neural and pupil terms cannot be taken as definitive evidence of independence. We have both softened the language around the claim (L368) and added a sentence to the Discussion (L464-468) acknowledging that this lack of correlation may reflect underlying noise and/or variability rather than true independence of the underlying mechanisms.

**Reviewer #1 (Recommendations for the authors):**

(3) The neural data analyses rely fundamentally on "switch" trials (Figures 3-5). It might be informative to also examine "non-switch" trials to see if there are specific neural markers indicating the exact moment the motion stimulus becomes behaviorally relevant. Given that this may fall outside the primary focus of the paper, it is up to the authors whether to pursue this line of inquiry.

We thank the reviewer for this suggestion. We agree and have added new analyses of data from non-switch trials (Extended Data Fig. 9), which show some effects of stimulus information from the adapting epoch on the monkeys’ choices, as we detail below in response to related comments from the other reviewers.

**Reviewer #2 (Public review):**

Aspects of the behavioral analysis would benefit from a tighter connection between theoretical claims about evidence accumulation and the empirical features of the psychometric functions. For example, the rightward shifts observed across adapting conditions are interpreted as consistent with a reset of accumulation on switch trials, but similar patterns could also arise from failures to detect the test stimulus on a subset of trials, leading responses to default to the final adaptor direction. Likewise, changes in psychometric slope and asymptote are attributed to differences in evidence accumulation without explicit modelling or consideration of alternative explanations.

Clarifying how specific features of the psychometric functions map onto distinct components of the decision process will strengthen the link between the theoretical framework and the behavioral data.

We agree and have made substantial revisions to address these important points. Specifically, we added a new figure panel (Fig. 2D), new Extended Data Figures (3 and 9), and several lines of explanatory text (L152-179) that collectively describe the behavior in more detail, including clarifying that: (1) for the shortest viewing durations, the monkeys’ decisions were informed by information from the adapting stimulus, which accounts for generally lower accuracy on LSF (longer exposure to the final adapting direction, thus more accumulated evidence for that direction before processing the switch) vs. HSF (shorter exposure to the final adapting direction, thus less accumulated evidence for that direction before processing the switch) switch trials; and (2) as viewing duration increased, the rate of rise of accuracy versus viewing duration was higher for LSF vs. HSF trials, implying differences in the process of evidence accumulation. As detailed in our response to a similar comment from Reviewer 1, above, we are now careful to temper our claims about the specific computational basis (e.g., a leak or other form of nonlinearity) for these differences.

We also de-emphasized our treatment of the asymptotes of the psychometric functions. In principle, these regimes could give insights into leakiness (which can limit the total amount of information that can be accumulated) and lapses (which are measured at the asymptotes). In practice, however, the long-duration trials that constitute the asymptotes were relatively under sampled (to promote the unpredictability of the offset of the stimulus, which we believed was the more important consideration when designing the experiment), yielding unreliable estimates.

A slight concern is the lack of a consistent analytical approach for relating behavioral changes to neural and pupil-linked measures. Different sections of the manuscript rely on different behavioral metrics-such as differences in accuracy within a selected stimulus-duration range (e.g., Figure 5C) or psychometric slope differences (Figure 6C) without clear justification for these choices. The analytical approach likewise varies between simple correlational analyses (Figure 5C, Figure 6C), pseudo-experimental group comparisons (Figures 5D, E), and the inclusion of neural or pupil terms in the behavioral psychometric regression model (Figure 7B). While each metric and approach may be defensible in isolation, adopting a more consistent framework will help convince readers that the reported effects are robust and not contingent on the selective choice of metric or analysis.

We thank the reviewer for this thoughtful critique and agree that the rationale for our choice of behavioral metrics and analytical approaches could be stated more clearly. We have added text to the relevant sections of the Results (L247-251) clarifying these choices. In particular:

The neural analyses (Figures 3D-E, Figure 4, Figure 5D-E) focused on preferred-motion switch trials, because: (1) low switch-frequency non-switch trials provide an additional 800 ms of exposure to the final adapting-stimulus motion direction relative to high switch-frequency non-switch trials, which confounds comparisons of context-dependent evidence encoding between conditions, and (2) MT neurons exhibit minimal responses to null motion (although note that we also included analyses based on ROC area, which is computed from both preferred- and null-motion switch trials, to account for possible contributions of null-motion responses; Figure 5A-C). Thus, to ensure a meaningful comparison between neural and behavioral measures, we used behavioral accuracy on switch trials as the relevant metric in Figure 5C-E, rather than psychometric slope, which is estimated across both switch and non-switch trials.

The pupil analyses (Figure 6) focused on a time window preceding test-stimulus onset, representing the arousal state around when the decision process started, and included both switch and non-switch trials. Thus, for these analyses we used psychometric slope, which is estimated across both switch and non-switch trials.

We used several different analyses to compare and contrast the neural-behavioral and pupil-behavioral relationships because they provide complementary and useful insights. The correlational analyses in Figures 5C and 6C characterize session-level relationships between neural/pupil signals and behavior. The group comparisons in Figures 5D–E provide a complementary visualization of the same relationship. The model-based approach in Figure 7 then allows direct quantification of the trial-wise contributions of each signal to behavior within a common framework. Importantly, the conclusions drawn from each approach converge on the same interpretation, which we believe speaks to the robustness of the reported effects.

**Reviewer #2 (Recommendations for the authors):**

(1) Figure 2 legend. Description of 'running average (5-trial window)' is unclear - presumably this is a running average in stimulus space rather than across trials.

We thank the reviewer for flagging this ambiguity. We have updated the legend (L136-137) to clarify that the running average is computed across trials sorted by test-stimulus duration.

(2) L158. Difficult to establish an asymptotic performance level for HSF conditions within the stimulus duration range tested.

We have removed the reference to asymptotic performance and replaced it with a discussion of performance on longer-duration switch trials in the context of the newly added Figure 2D.

(3) L515 Equation 1. While this is a standard formulation of lapse rate in psychometric functions, the construction here in terms of switch probability is not standard. Given the task and training, it seems more likely that on lapse trials, the animal will respond according to the last adapted direction (rather than randomly switch/stay with equal probability).

We thank the reviewer for this point. We agree that it is possible that on at least some of the “lapse” trials the monkeys may respond according to the final adapting-stimulus direction rather than choosing randomly. However, we cannot distinguish those alternatives using this task design. We include a statement to this effect in Methods (L569-571).

To explore the idea further, we refit the behavioral data using separate upper and lower asymptotes corresponding to lapse rates on switch and non-switch trials, respectively. Across monkeys, there were no significant differences between upper and lower lapse rates for either low (Wilcoxon signed-rank test for equal medians: *p* = 0.15, Cohen's *d* = -0.13) or high switchfrequency (*p* = 0.07, Cohen's *d* = -0.16) conditions. So, at the very least, there was no evidence for lapse-like errors driven by switch- (or non-switch-) specific defaults to the final adapting direction.

(4) L256. Statistical significance of attenuation is not directly tested here.

We have replaced "were attenuated" with "we did not identify any reliable context-stability differences" (L297) to accurately reflect what was directly tested without implying a statistical comparison between groups of sessions that was not performed.

(5) L429. Does the increase in explanatory power warrant the increased complexity of the model here?

We thank the reviewer for raising this important point. We used Tjur's pseudo-R^2^ because it does not increase by default with added model complexity, making it more conservative than other R^2^ measures in this respect. Tjur's pseudo-R^2^ is a coefficient of discrimination, and as such its value increases only when additional terms improve the model's ability to separate predicted probabilities across response outcomes. Thus, the observed increases in explanatory power when adding neural or pupil terms reflect real improvements in discriminability rather than an artifact of model complexity. We have added a brief clarification of this point to the Methods (L662-664).

**Reviewer #3 (Public review):**

The task design may not be optimal. While the amount of time the monkey is exposed to each motion direction during the adapting stimulus is matched, it's hard to know if the reduced MT responses to the test stimulus are truly due to the greater frequency of switches during the HSF adapting stimulus or because the monkeys have been exposed to more repetitions of the stimulus. It's increased sensory adaptation in either case, but it makes it problematic to interpret this as temporal context-dependent adaptation specifically. I think this could potentially be partially addressed by an analysis that is in the paper, but could potentially be emphasized/fleshed out more, specifically the results shown in Figure 4D that seem to show that most of the reduction in neural response for adapting units occurs between the first and second stimuli.

The reviewer raises an important point. The number of stimulus repetitions and switch frequency are confounded in the experimental design, making it difficult to attribute context-dependent differences in MT responses to the temporal pattern of switches rather than to accumulated repetitions. We also note, as the reviewer acknowledges, the observed differences reflect sensory adaptation either way. Figure 4D does offer relevant evidence, suggesting that a majority of the change in neural response occurred with just one stimulus repetition. This finding complicates an interpretation where adaptation scales with the number of stimulus repetitions. We have added several lines to the Results about these points (L231-233).

The pupillometric analysis seems to be an indirect way of assessing whether the accumulator itself might be modulated by temporal context, but the link could be made clearer. The authors show that context-dependent behavior is related to pupil size, which is related to arousal/neuromodulation, but it would be helpful to have some idea of what neural mechanisms underlying adaptive decision-making are actually impacted by this neuromodulation. Lacking neural data to address this question (e.g., from a brain region proposed to be involved in the accumulation process), at least more discussion of this would be helpful. Essentially, I'm unsure of how to interpret the pupil results: the argument that temporal context affects instantaneous evidence encoding in MT that then drives the accumulator is very clear, but I am a bit confused about what, mechanistically, I should think about the effect of neuromodulation doing.

We thank the reviewer for this thoughtful comment and agree that the mechanistic interpretation of the pupil results could be made clearer. We acknowledge that we cannot directly identify the neural mechanisms underlying the arousal-related contributions to adaptive evidence accumulation from pupil data alone, given that pupil size is an indirect and imperfect proxy for neural (e.g., LC-NE system) activity. However, we can offer some informed conjecture and have added to the Discussion (L469-482) in an effort to elaborate on possible mechanisms.

**Reviewer #3 (Recommendations for the authors):**

(1) Abstract could be retooled - does not emphasize the pupillometry/arousal results very much, and they are presented more as a control than an independent result.

We agree and have revised the Abstract accordingly.

(2) Do all neural/pupil analyses use only switch trials? Sometimes the figure captions do specify only switch trials, but not everywhere. It would be helpful to specify either in the Methods or at the beginning of each figure caption that all subplots show switch trial results. Also, if you do always use switch trials, it would be useful to see in the Supplement how the non-switch trial results differ from switch trials. It seems like they may in interesting ways based on the behavioral results (supporting a reset of evidence accumulation on switch but not non-switch trials).

We thank the reviewer for flagging these important points. We have added a justification for switch trials (L186-190) as well as clarification about which trial types were used for which analyses (L246-249) and information about trial types to relevant figure captions. We have also added a new Extended Data figure (Extended Data Fig. 9) examining relationships between neural activity and behavior on non-switch trials. As inferred by the reviewer, behavior on non-switch trials is consistent with the use of information from the adapting stimulus.

(3) In Figure 3C, 5B, etc, when computing firing rate for the test stimulus (50-500 ms), are differently sized windows used to compute the rate for different test stimulus durations (since some will be <500 ms)? Or are only trials where the test stimulus duration is > 500 ms used for this analysis?

We thank the reviewer for raising this point. To clarify, the 50–500 ms window does not reflect a fixed window applicable for all trials. Rather, neural activity from 50 ms after test-stimulus onset through test-stimulus offset was included for each trial, with 500 ms serving as the upper bound for trials with longer durations (> 500 ms). We have clarified this in the Methods (L607-610) to avoid ambiguity.

(4) I think it might be better to be consistent with the time windows used for analysis; specifically, to choose either the 50-500 ms window used in Figures 3, 4, and 5B, or the 200- 400 ms window used for the remaining analyses in Figure 5.

We agree that using the same window for all of the analyses would improve consistency, but not doing so provides advantages that we believe take precedent and now describe in more detail. The broader 50–500 ms window used for Figures 3, 4, and 5B was chosen to characterize MT neural activity over a relatively large a time window, ensuring that every trial contributes to each estimate. Because test-stimulus durations were drawn from a truncated exponential distribution (100–1200 ms), restricting these analyses to the 200–400 ms window would have excluded the substantial proportion of trials with durations <200 ms (but would yield similar figures and conclusions). The narrower window used in subsequent analyses allows us to focus on the conditions that exhibited the biggest modulations of neural activity when comparing them to behavior.

(5) Similarly, provide justification for using only trials ending 375-600 ms after test stimulus onset for the behavioral correlations. It seems reasonable to choose a subset of test stimulus durations where the monkeys' behavior is greater than chance but less than ceiling, but it would be good to specify this so that it doesn't seem arbitrary.

We agree and have added text to make this important point (L249-251).
